# Maternal obesity and its relation with the cesarean section: A hospital based cross sectional study in Iraq

**DOI:** 10.1186/1471-2393-14-235

**Published:** 2014-07-17

**Authors:** Waqar Al-Kubaisy, Mazin Al-Rubaey, Redhwan A Al-Naggar, Ban Karim, Nor Aini Mohd Noor

**Affiliations:** 1 Population Health and Preventive Medicine Department, Faculty of Medicine, Universiti Teknologi MARA (UiTM), Selangor, Malaysia; 2 Community Medicine Department, Faculty of Medicine, AL-Mustanseryah University, Baghdad, Iraq; 3 Drug Discovery & Health Community of Research, Universiti teknologi MARA (UiTM), 40450 Shah Alam, Selangor, Darul Ehsan, Malaysia; 4 Humanity and Quality of life, Universiti teknologi MARA (UiTM), 40450 Shah Alam, Selangor, Darul Ehsan, Malaysia

**Keywords:** Obesity, Cesarean section, Route of delivery, Iraq

## Abstract

**Background:**

Obesity during pregnancy is reported in approximately one in five pregnant women worldwide. It increases the risk of pregnancy complications many of which necessitate Cesarean section (CS). This study determines the association between obesity and type of delivery.

**Methods:**

A cross-sectional study involving 404 pregnant women was carried out at Al-Yarmouk Teaching Hospital, Baghdad, Iraq. Women with hypertension, diabetes, preterm labor, fetal presentation other than cephalic presentation and multiple gestations were excluded from the study. BMI and past obstetric history were recorded.

**Results:**

The overall rate of CS was 38%. The overall mean body mass index (BMI) was 25.0 ± 4.52 Kg/m^2^ and it was significantly higher among women who delivered by cesarean section. Significantly high rate of CS was found in primigravida and multigravida women with high BMI. Moreover, all obese multigravid women with history of previous CS were delivered by CS. The rate of CS was higher in women with primary level education when compared to women with secondary or tertiary education. CS was significantly lower in women with a previous history of abortion.

**Conclusion:**

Obese women with or without a previous history of CS are at a higher risk of having a CS and should therefore be considered as high risk and managed appropriately during pregnancy. Weight management prior to or during pregnancy could help reduce the need for CS.

## Background

Obesity is most commonly measured as a weight to height ratio and expressed as body mass index (BMI) [1]. It is an internationally accepted method that provides a reliable way to assess obesity related health problems. The prevalence of obesity in adults is increasing worldwide, particularly among women of child bearing age. In Iraq, according to the Ministry of Health report the prevalence of obesity in women in reproductive age was 38.2% in 2006 [2]. Moreover in Baghdad between 1997 and 2007 the prevalence of obesity among women in reproductive age increased from 23.6 to 25% [3,4]. Globally the prevalence of maternal obesity has also increased, rising from 9-10% in the early 1990’s to 16 - 19% in the year 2000 [5-7]. In Dublin, 19% of women in their 1^st^ trimester were obese [8]. Similarly in US, obesity among pregnant women ranged from 18.5 -38.3% [9]. Maternal obesity is defined as women who have a BMI ≥30 Kg/m^2^ at first antenatal visit [5]. It is calculated by dividing pre-pregnancy weight in kilograms (Kg) by height in meter square (m^2^) [6]. It is evident that obesity increases the risk of pregnancy complications. In obese mothers, the perinatal morbidity such as pre-eclampsia and gestational diabetes is high [10-12]. Obesity is associated with increased rates of cesarean section (CS) [12]. Several recent reports document rates of CS 32% in USA [10], 19.1% in Saudi Arabia [11], 9.29% in Jordan [13] and 20.1% in Iraq [14]. Interestingly also the rates of CS in Baghdad city were 17.9, 19 and 28.7% in 2007, 2009 and 2011; respectively [15,16].

Dietz *et al*. (2005) showed that the rate of cesarean section was 14.3% in lean gravid women and 42.6% in obese women [17]. A linear relationship between BMI and cesarean delivery has been reported [18]. Obese women were 6 times more likely to have cesarean section due to cephalo-pelvic disproportion or failure to progress than non-obese women [19].

Earlier studies in Iraq have not evaluated the association of maternal obesity with other factors like parity and previous cesarean section, which are known risk factors for cesarean section. Therefore, the aim of this study was to determine the association of increased BMI among primigravid, multigravida with and without history of CS with the mode of delivery in the current pregnancy.

## Methods

A cross-sectional study was conducted at Obstetrics Department of Al-Yarmouk Teaching Hospital, Baghdad, Iraq from January to May 2012. Ethical approval was obtained from the Ethics Committee at Ministry of Health, Baghdad, Iraq. A convenience sample of 404 pregnant women attending the labor room with induced or spontaneous labor was collected. Informed consent was obtained from each participant. The inclusion criteria were women with normal pregnancy (no pathological conditions) at ≥ 37 weeks of gestation. Primigravida and multigravida with normal pregnancy and live cephalic singleton fetuses were included. Pregnant women with elective cesarean section, hypertension, and diabetes were excluded. In addition, multiple gestations, and abnormal lie fetus at time of labor were also excluded. Each participant was interviewed using a well constructed questionnaire. The questionnaire include socio-demographic (age, education and husband’s occupation) and obstetrical (parity, history of abortion, type of previous delivery) information. In addition, height and weight were measured and the pre-pregnancy weight was calculated by subtracting 12.5 Kg from the current weight. The average weight gain during pregnancy is estimated as 12.5 Kg [5]. Statistical analysis was done using tests contained in SPSS 20. Descriptive data are presented in frequencies, simple percentages. Chi-square (x^2^) test was used to evaluate the associations between categorical variables. ANOVA was used to test the significance of differences between more than two means and Z test was used to test the significance of difference of two means. A p-value of < 0.05 was considered significant.

## Results

A total of 404 women were included in this study. More than half of them (58%) were 20-30 years old and more than two thirds (68%) had less than secondary school education. Out of 404 women 154 (38.2%) their current pregnancy were ended by CS. Significant association between mother’s education and type of delivery (p = 0.036) was present. Women with lower primary education had significantly higher rate of CS (41.6%) than those with secondary school and above education (30.8%) (p = 0.036). Women with history of previous abortion showed significantly lower rate of CS compared to women with no history of abortion (p = 0.04). However, this study showed that the mode of delivery was neither associated with the age of the women nor with the occupation of the husband (p = 0.324, p = 0.191; respectively). Regarding the relationship between gravidity of the mother and mode of delivery, multigravidae with previous history of CS had a significantly higher rate of CS (58.5%) in the current pregnancy when compared to other two groups (p < 0.001) (Table 1). The overall mean BMI in the study subjects was 25.05 ± 4.25 kg/m^2^ (ranging from 16.2 to 39.1) (Table 2). More than half (51%) of the participants were within the normal range of BMI. However, 45.3% were overweight and obese (Table 3). Significant variation in mean BMI was detected among mothers with different gravidity (Table 2). The highest mean BMI was among the multigravidae with a previous history of CS (25.77 ± 4.34 Kg/m^2^), while primigravidae had the lowest mean BMI (24.31 ± 4.41; p = 0.029) (Table 2). Studying the relationship between mode of delivery in the current pregnancy and BMI level, we gave an evidence that pregnant women whom their pregnancy ended by CS, their BMI was (28.62 ± 4.49) significantly higher (p < 0.001) than (22.85 ± 2.82) those whom delivered normally (Table 2). Moreover, with increasing BMI (≥25) above normal, CS was significantly increased steadily (p < 0.001) (Table 3). All obese (BMI ≥ 30) multigravidae with a previous history of CS were delivered via CS. CS for the current pregnancy was significantly higher among obese (BMI ≥ 30) primi and multigravida women without previous history of CS (85.7% and 78.3% respectively; p < 0.001) compared to non-obese primagravidae and multigravidae (28.1% and 15.9%, respectively) (Table 4).

**Table 1:** Characteristic profile of Iraqi pregnant women based on the mode of delivery (n = 404)

**Variable**	**Total**		**NVD**		**CS**		**χ**^**2**^	**P value**
		**No**	**%**	**No**	**%**	**No**	**%**	
Age (years)								
<20	50	12.4	30	60.0	20	40.0	4.661	0.324
20—24	134	33.2	83	61.9	51	38.1		
25—29	100	24.8	67	67.0	33	33.0		
30—34	82	20.3	52	63.4	30	36.6		
≥35	38	9.4	18	47.4	20	52.6		
Husband occupation								
Non-governmental	238	58.2	141	59.2	97	40.8		1.17	0.191
Governmental	166	41.1	109	65.7	57	34.3			
Women’s level of education									
Less than Secondary	274	67.8	160	58.4	114	41.6		4.39	0.036
Secondary and higher	130	32.2	90	69.2	40	30.8			
History of Abortion									
No	373	92.3	225	60.3	148	39.7		4.19	0.04
Yes	31	7.7	25	80.6	6	19.4			
Pregnant women classifications									
Primigravida	149	36.9	95	63.8	54	36.2		28.9	<0.001
Multigravida without CS	149	36.9	111	74.5	38	25.5			
Multigravida with CS	106	26.2	44	41.5	62	58.5			

**Table 2:** Mean BMI of 404 Iraqi pregnant women in relation to route of delivery

**Variable**	**No**	**Mean ± SD**	**Test of significance**	**p value**
All women	404	25.05 ± 4.25		
Primigravidae	149	24.31 ± 4.41	F = 3.562	0.029
Multigravidae without CS	149	25.27 ± 4.67		
Multigravidae with CS	106	25.77 ± 4.34		
Current pregnancy with				
NVD		22.85 ± 2.82	Z = 15.848	<0.001
CS		28.62 ± 4.49		

**Table 3:** Mode of delivery of Iraqi pregnant women and BMI (n = 404)

**Variable**	**Total**		**NVD**		**CS**		**χ**^**2**^	**P value**
	**N**	**%**	**N**	**%**	**N**	**%**		
BMI								
Underweight (<18.5)	15	3.7	13	86.7	2	13.3	180.1	<0.001
Normal weight (18.5-24.9)	206	51.0	187	90.8	19	9.2		
Overweight (25-29.9)	116	28.7	42	36.2	74	63.8		
Obese (≥30)	67	16.6	8	11.9	59	88.1		

**Table 4:** Gravidity and mode of delivery based on BMI (n = 404)

**Gravidity**	**BMI < 30**		**BMI** ≥ **30**		**χ**^**2**^	**p value**
	**NVD**	**CS**	**NVD**	**CS**		
Primi gravid	92 (71.9%)	36 (28.1%)	3 (14.3%)	18 (85.7%)	25.89	<0.001
Multigravida without CS	106 (84.1%)	20 (15.9%)	5 (21.7%)	18 (78.3%)	39.85	<0.001
Multigravida with CS	44 (53%)	39 (47%)	0	23 (100)		

## Discussion

In this study many factors “for example diabetes mellitus, hypertension, fetal presentation other than cephalic and multiple pregnancies” that may relate to CS were excluded. In spite of that, the rate of CS delivery was higher than (22.6%, 33.3%) that reported previously in Iraq [16,20], Saudi Arabia [11], Jordan [13], USA [10] and Pakistan, where it was reported as 32.3% [21]. Moreover, mean BMI of the pregnant women who delivered via cesarean section was significantly higher than those via vaginal delivery. In addition, there was a significant positive association between BMI and the rate of CS. As BMI increased the rate of CS too increased, suggests that the high rate of CS in our study is mostly due to the high BMI of these women. Our finding is in agreement with Kominarek's *et al*., (2010) in US [22] who found that cesarean deliveries increased significantly across the different classes of obesity. Similarly, Tosson and Al-hussaini in Egypt, (2005) [23] as well as Perlow *et al*., (1992) [24] suggested that BMI was significantly related to the mode of delivery. Cnattingham *et al*., (1998) in reported that obese women due to their large body volume, more time may be taken for oxytocin to reach the optimal tissue level [25]. Also presence of excess intra abdominal adipose tissue itself could mechanically obstruct the progression of labor, this could, overtime, compromise fetoplacental circulation and cause fetal distress and necessitating CS. Although higher rate of CS was found among women aged 35 years and above, this finding was however not significant, which is in contrast to the findings of Perlow *et al*., (1992) [24].

Our findings are also in agreement with an earlier study [26], where it was reported that women with high BMI and previous history of CS are less likely to have normal vaginal delivery during the next pregnancy. In this study it was noted that none of those obese (BMI ≥ 30) women with a previous history of CS had ended by normal vaginal delivery. Moreover, a significantly high proportion of obese (BMI ≥ 30) primigravida or multi gravidae with no history of CS in this study had their current pregnancy ended by CS. The significantly higher rate of cesarean section among women with lower educational level supports the findings of another study in Baghdad [27]. The possible explanation is that the women with higher education are more likely to take care of their weight and body shape and practice a healthy lifestyle such as regular exercise and healthy diet. Another explanation is that low educational level may lead to poor utilization of health services during pregnancy.

## Conclusion

This study showed a significant association between BMI and increased risk of cesarean delivery. Obese women should therefore be considered as high risk and managed appropriately during pregnancy. Weight management should be implemented in the primary care clinics to counsel women to reduce their weight.

## Competing interests

The authors declare that they have no competing interests.

## Authors’ contribution

WA study design and writing the manuscript. MA study design, obtaining ethical approval from Ministry of Health, Iraq, data collection and writing the manuscript, RA statistical data analysis and writing the manuscript. BK data collection. NA agreed to be accountable in all aspects of the work, data interpretation, critical revision of the manuscript, final approval and final editing. All authors read and approved the final manuscript.

## Pre-publication history

The pre-publication history for this paper can be accessed here:

http://www.biomedcentral.com/1471-2393/14/235/prepub
